# Tropical Estuarine Macrobenthic Communities Are Structured by Turnover Rather than Nestedness

**DOI:** 10.1371/journal.pone.0161082

**Published:** 2016-09-01

**Authors:** Carlinda Raílly Medeiros, Luiz Ubiratan Hepp, Joana Patrício, Joseline Molozzi

**Affiliations:** 1 Postgraduate program in Ecology and Conservation, State University of Paraíba, Campina Grande, Paraíba, Brazil; 2 Department of Biological Sciences, Integrated Regional University of Alto Uruguai and Missões, Erechim, Rio Grande do Sul, Brazil; 3 MARE–Center for Marine and Environmental Sciences, School of Sciences and Technology, University of Coimbra, Coimbra, Portugal; 4 Department of Biological Sciences, State University of Paraíba, Campina Grande, Paraíba, Brazil; Auckland University of Technology, NEW ZEALAND

## Abstract

Turnover (i.e., species substitution) and nestedness (i.e., subsets of species from more diverse locations), the two main mechanisms used to explain the beta diversity of biological communities, have different implications for biodiversity conservation. To better understand how these mechanisms contribute to beta diversity, we tested the following hypotheses: (i) greater dissimilarity in community composition occurs between estuarine zones than other hierarchical level studied; (ii) beta diversity in these communities develops by turnover in estuaries with a lower degree of anthropogenic impact, but by nestedness in estuaries with a greater degree of anthropogenic impact; and (iii) the structuring mechanism is independent of season. We studied two tropical estuaries (dry and wet seasons) that vary in terms of land-use of the drainage basins. Subtidal benthic macroinvertebrates were sampled along the estuarine gradient in each of the two estuaries. The additive partitioning approach to species diversity was used to determine the hierarchical scale with the greatest dissimilarity in community composition. General beta diversity was measured using the Sorensen dissimilarity index, partitioning the turnover and nestedness components. The greatest dissimilarity in the composition of the communities occurred between the zones along the estuarine gradient in both seasons (dry = 58.6%; wet = 46.3%). In the estuary with a lower degree of anthropogenic influence, benthic macroinvertebrate diversity was generated by turnover regardless of the season. In the estuary with a greater degree of anthropogenic impact, beta diversity was structured by turnover during the dry season and a combination of both mechanisms during the wet season. We conclude that turnover is the principal mechanism responsible for beta diversity in benthic macroinvertebrate communities in tropical estuaries.

## Introduction

Understanding the composition and distribution of biological communities in relation to environmental conditions on a local or regional level is one of the main challenges facing ecologists [1]. Partitioning diversity allows a better comprehension of the mechanisms that guide the structuring of communities along environmental gradients and over spatial scales [2]. An understanding of the dominant structuring mechanisms has important implications for biodiversity protection, since conservation strategies are more effective if these mechanisms are considered.

The total diversity of species in a given region (gamma, γ) is composed of alpha and beta components [3], which can vary along spatial, temporal or environmental gradients [4, 5]. Alpha (α) diversity corresponds to local diversity and can be estimated by species richness or diversity indices [6]. Beta (β) diversity is a measure of dissimilarity (i.e., variability) in community composition between areas [7]. The scientific community has focused his studies on beta diversity using the additive partitioning approach. This approach allows us to understand the spatial scales that generate variations in community composition and affect the formation and evolution of biological communities in different environments [8, 9]. Variations (dissimilarities) in the composition of benthic communities in different areas are mainly caused by factors based on niches (e.g., the adaptation of species to different habitats) or environmental aspects (e.g., salinity) and factors related to geographical distances (e.g., propagule dispersal) [10]. The influence of factors that generate dissimilarity in the composition of communities results in either the substitution of species (turnover) or the formation of subsets of more diverse sites (nestedness). The relative importance of the dissimilarity mechanism varies in accordance with the spatial scale considered and temporal changes [11, 12].

Limnological factors acting on a local scale (e.g., dissolved oxygen and salinity) can give rise to variations in community composition through species selection [13]. Environmental conditions serve as filters selecting for intrinsic functional attributes of species, defining which species can persist as community members and reproduce in a given habitat, so that only those with adequate phenotypic characteristics for local environmental conditions pass through these filters [14, 15]. As this filtering process affects the biological attributes of species, habitats with similar characteristics should have pools of species with similar functional and morphological characteristics that are selected in accordance with the spatial and temporal variations in local environmental conditions [16].

At large scales, such as landscape or eco-region can limit the species dispersal [7, 1]. When dispersal is reduced, the dynamics of organisms are limited and the community is spatially distributed along a gradient [17, 18]. Thus, the composition and structure of communities on a regional scale may reflect environmental filters and are regulated by the dispersal capacity of species [17, 19, 20]. The passive dispersal of benthic invertebrates in marine and estuarine systems is a fundamental process influenced by water movements due to continental drainage and tides [21]. Under favorable environmental conditions, dispersal allows the maintenance of species in different areas through migration [13].

Beta diversity can be due to two mechanisms, turnover and nestedness [21–23]. These two components are regulated by the limnological conditions of systems that control species distribution. Turnover regards the directional substitution of some species by others, with either an increase or decrease in the number of species along spatial, temporal or environmental gradients [23]. Nestedness occurs when sites with a lower degree of species diversity constitute a subset of biologically more diverse sites [21]. These components are diametrically opposed and equilibrium is established between the substitution and nestedness of species in response to different types and intensities of environmental impact [24, 25].

Benthic macroinvertebrates in estuarine communities are adapted to the available ecological habitats and niches where the community composition vary depending on the dynamics of the structures the community and functioning of these ecosystems [26–29]. Estuaries are transition systems between rivers, which transport fresh water, and the ocean, which is responsible for the influx of salt water stemming from tidal action. The dynamics of estuaries leads to the establishment of an environmental gradient expressed by gradual changes in salinity as well as variations in the composition and granulometry of the sediment and the amount of available organic matter [29–32]. The high environmental variability of estuaries naturally stresses these ecosystems and the dynamism of environmental factors guides the distribution of benthic macroinvertebrate species [33]. Together with these natural stressors, estuaries are also subjected to a high degree of anthropogenic impact, which also exerts an influence on the distribution of species [25].

Generally, in estuaries with low anthropogenic influences, the observed environmental changes are not strong enough to exclude the non-tolerant species to pollution. In this situation, a constant modification occurs (replacement) in species that make up the communities, called turnover [24, 25]. In contrast, in estuaries subjected to a greater degree of anthropogenic influence, species are strongly affected by this environmental variation, resulting in the elimination of organisms. Thus, in these sites species diversity consists mainly of general organisms tolerant to environmental disturbances. It is possible that such species are present throughout the entire estuarine gradient, including areas with greater species diversity, resulting in nested subsets of species [25].

The difference in community composition is the result of the action of different mechanisms (e.g. turnover and nestedness) that cause beta diversity [23]. Differentiating the components of beta diversity (turnover and nestedness) results in different implications with regard to the establishment of priority areas for the conservation of biodiversity [34]. When beta diversity is governed by nestedness, this pattern can be observed by the presence of generalist species throughout the estuarine gradient (including areas with greater diversity) that resisted the environmental filters that acted in the selection of species. Thus, conservation strategies should give priority to areas with greater species diversity [35,36]. In sites with a low diversity, measures should be developed to mitigate environmental impacts and create better conditions to expand local diversity. For cases in which communities are structured by turnover, however, various sites should be the target of conservation [35,36].

The aims of the present study were to evaluate the additive partitioning of the benthic macroinvertebrate community in tropical estuaries, determine whether beta diversity in benthic communities is structured by turnover or nestedness and determine whether the observed structuring patterns are constant and independent of season. For such, the following hypotheses were tested: (i) greater dissimilarity in community composition occurs between estuarine zones than other hierarchical levels studied; (ii) the beta diversity of the benthic macroinvertebrate community in an estuary with a low degree of anthropogenic impact is generated by turnover due to slight influence of environmental variation on community composition, while beta diversity in an estuary with a high degree of anthropogenic influence is generated by nestedness due to the formation of subsets of generalist species; and (iii) structuring patterns are independent of seasonal changes, as the mechanisms of turnover and nestedness are principally related to the intensity of anthropogenic influences.

## Materials and Methods

### Study area

**Fig 1:**
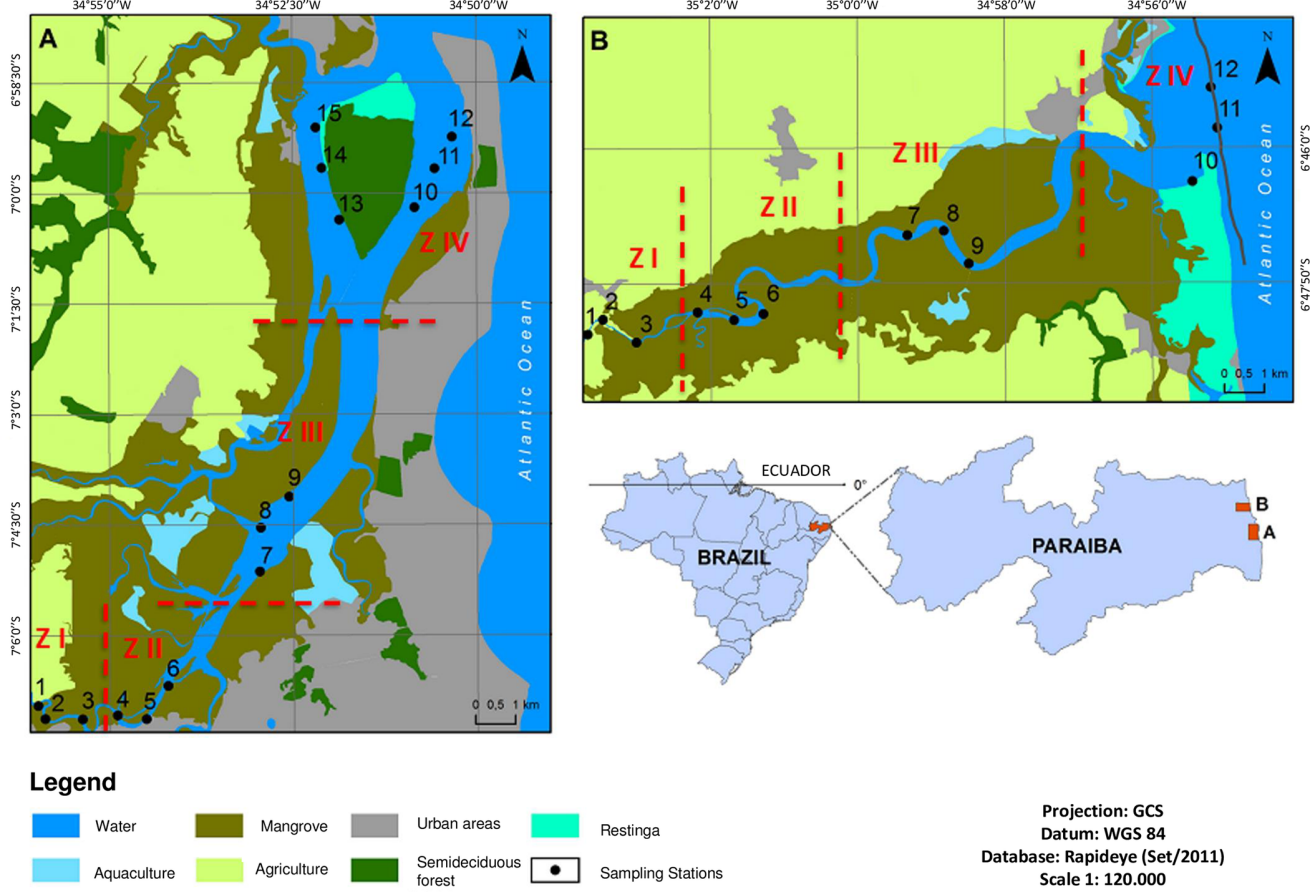
Location of Paraíba do Norte (A) and Mamanguape (B) estuaries in state of Paraíba, Brazil, and sampling zones defined as I, II, III and IV.

Two estuaries were selected to test the hypotheses: Paraíba do Norte (6°54’14” - 7°07’36”S; 34°58’16” - 34°49’31”W) and Mamanguape (6°43’02” - 6°51’54”S; 35°67’46” - 34°54’04”W), both of which are located in the tropical region of Brazil (Fig 1). The regional climate is hot and humid (type As’ according to the Köppen system) [37], with air temperature ranging from 25 to 30°C. The wet season occurs from February to July and the dry season is October to December [38]. The months of January, August and September correspond to transition periods between the wet and dry seasons. The estuaries differ principally in terms of their use and occupation by human populations (Fig 1).

The Paraíba do Norte estuary (Fig 1A) is bordered by urban agglomerations with a total population of approximately 1,100,000 inhabitants [39]. This estuary is under the direct influence of urban activities and receives domestic waste [40]. The estuary covers 22 km [41] and is influenced by inputs from the Atlantic Ocean, especially in the dry season, due to the low flow of perennial contributing rivers. The Mamanguape estuary (Fig 1B) is located within the Barra de Mamanguape Environmental Protection Area (IUCN conservation category V), which was created in 1993 to protect a mosaic of habitats, including the Atlantic Forest, mangrove forests, coastal reefs, shoal vegetation, dunes and coastal cliffs, as well as the West Indian manatee (*Trichechus manatus* Linnaeus, 1758) and its natural habitats. This estuary extends 24 km and is surrounded by small towns with a total population of approximately 66,000 inhabitants. The mangrove forests surrounding the estuary are well preserved despite neighboring anthropogenic activities [42]. However, extensive sugarcane plantations are found in the surrounding area and the estuary received effluents from shrimp farming activities near the Gamboa River until 2012.

### Sampling method

Four upstream to downstream subtidal habitats (zones I, II, III and IV) of the Paraíba do Norte and Mamanguape estuaries were defined based on salinity, sediment granulometry and depth during a pilot study undertaken in August 2013 (Fig 1). Three sampling sites were established in each zone and three sampling points were established at each site [Mamanguape: points 1 to 3 (zone I), points 4 to 6 (zone I), points 7 to 9 (zone III) and points 10 to 12 (zone IV); Paraíba: points 1 to 3 (zone I), points 4 to 6 (zone I), points 7 to 9 (zone III) and points 10 to 15 (zone IV)]. The sampling points in each zone had similar sediment granulometry, salinity and depth.

**Fig 2:**
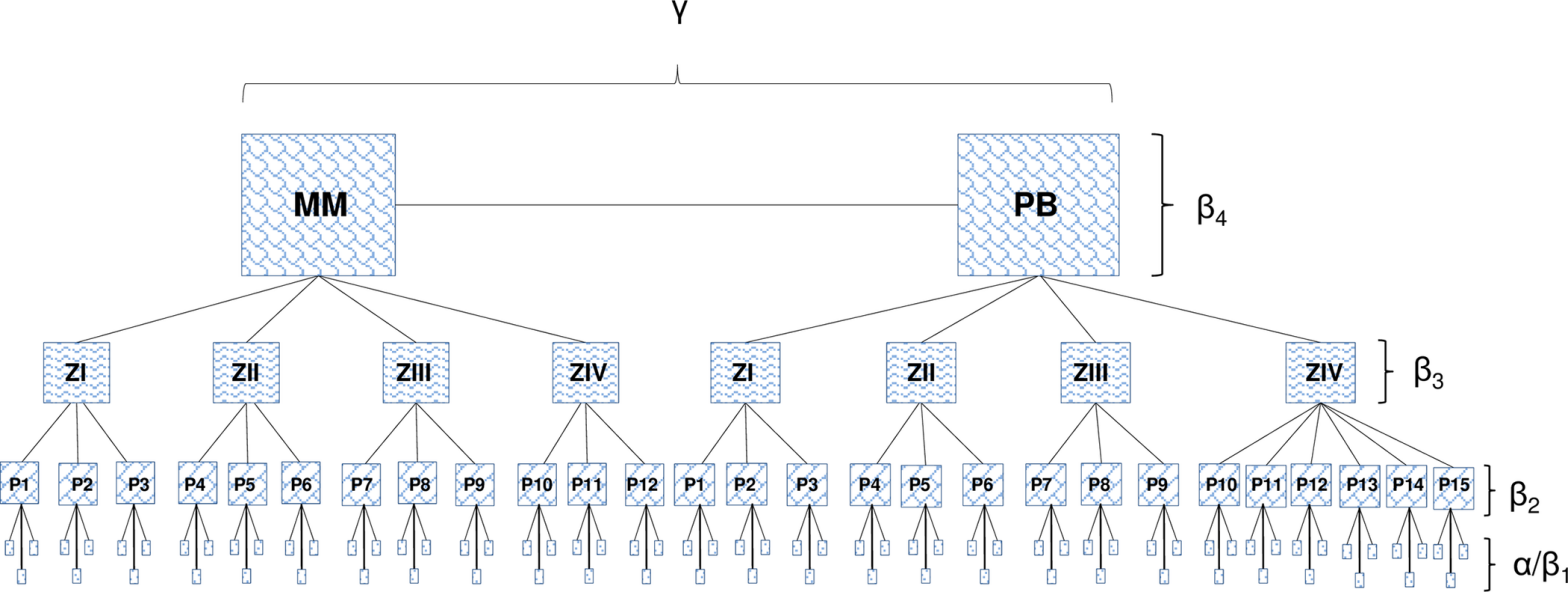
Hierarchical design used in macroinvertebrate sampling in Mamanguape (MM) and Paraíba do Norte (PB) estuaries as well as respective zones (ZI, ZII, ZIII, ZIV) and sampling points (Mamanguape: P1-P12; Paraíba do Norte: P1-P15).

This study was hierarchically delineated such that alpha diversity (α) corresponded to diversity within the sampling units, followed by levels that corresponded to variations in diversity among the sampling units (β_1_), variations in diversity among sampling points (β_2_), variations in diversity among zones (β_3_) and variations in diversity between estuaries (β_4_) (Fig 2).

### Sampling of benthic macroinvertebrates

Sampling of subtidal benthic macroinvertebrates was undertaken in November 2013 (dry season) and July 2014 (wet season). Benthic macroinvertebrates were collected using a van Veen grab sampler (0.1 m^2^). The collected sediments were preserved *in situ* in 4% buffered formalin and subsequently washed in sieves (0.5 mm mesh) in the laboratory. In the present study, only the Polychaeta, Mollusca, and Diptera groups were analyzed, which were identified on the genus or species level based on taxonomic keys for each group [43–48].

The macroinvertebrates collections in the Paraíba do Norte and Mamanguape estuaries were authorized by a permanent license of the Chico Mendes Institute for Biodiversity Conservation and Authorization System and Information on Biodiversity (ICMBio/SISBIO) #31000–1, both are Brazilian government institutes. The animals sampled in this study did not involve threatened or protected species.

### Data analyses

Permutational multivariate analysis of variance (PerMANOVA, with 9999 permutations; α ≤ 0.05) was used to evaluate differences in the composition of the benthic macroinvertebrate communities in the different seasons, estuaries and zones. The analytical design considered three factors: season (dry and wet), estuary (Paraíba do Norte and Mamanguape) and zone (zones I, II, III and IV). Abundance data were log (x+1) transformed and the Bray-Curtis index was used as a measure of similarity. Analysis of variance (three-way ANOVA; α ≤ 0.05) was used to evaluate differences in taxonomic richness among the three factors: season, estuary and zone.

A hierarchical scheme was used to evaluate the additive partitioning of diversity (Fig 2). The additive partitioning approach consists of comparing variability between hierarchical levels and producing direct percentage values [49]. An individual-based null type model was used to determine whether the components of observed diversity differed from the random diversity that would be expected if the individuals were randomly distributed among spatial scales [49]. The significance of each hierarchical level was determined by differences between the expected values and values obtained from 9999 permutations [49]. High proportions (Prop_exp>obs_ > 0.975) indicated that the observed values were lower than those expected randomly and low proportions (Prop_exp>obs_ < 0.025) indicated that the observed values were higher than those expected randomly. Regional diversity (γ) was obtained by the sum of α and β components (γ = α + β_1_ + β_2_ + β_3_+ β_4_).

The spatial patterns of beta diversity were determined by measuring dissimilarities among multiple sites randomly selected from a presence/absence matrix [50]. The general beta diversity of the estuaries was measured in the dry and wet seasons using the Sorensen dissimilarity index (βsor), which was partitioned into turnover (βsim) and nestedness (βnes) components. It was thus possible to evaluate whether dissimilarities in the composition of the biological community occurred through the substitution of some species by others (βsim) or the formation of nested subsets of more diverse communities (βnes) [23].

All analyses were performed with the aid of the R software [50] using functions of the “vegan” [51] and “betapart” [52] packages, except additive partitioning, for which the Partition 3.0 program [53] was used.

## Results

### Abundance, richness and community composition

The benthic macroinvertebrate communities (considering only Polychaeta, Mollusca and Diptera) sampled in the Paraíba do Norte and Mamanguape estuaries were composed of 24,438 individuals distributed among 130 taxa. A total of 17,407 individuals were recorded in the dry season and 7031 individuals were recorded in the wet season in the two estuaries. The composition of the communities in both estuaries differed between seasons (PerMANOVA: F_1,46_ = 3.23; p = 0.001). In both periods, the benthic macroinvertebrate communities were represented by larger proportions of *Polypedilum* (Chironomidae: dry = 62.1%; wet = 41.2%), *Laeonereis* (Polychaeta: dry = 24.9%; wet = 13.8%) and *Anomalocardia brasiliana* (Gmelin, 1791) (Mollusca: dry = 1.8%; wet = 4.0%).

**Fig 3:**
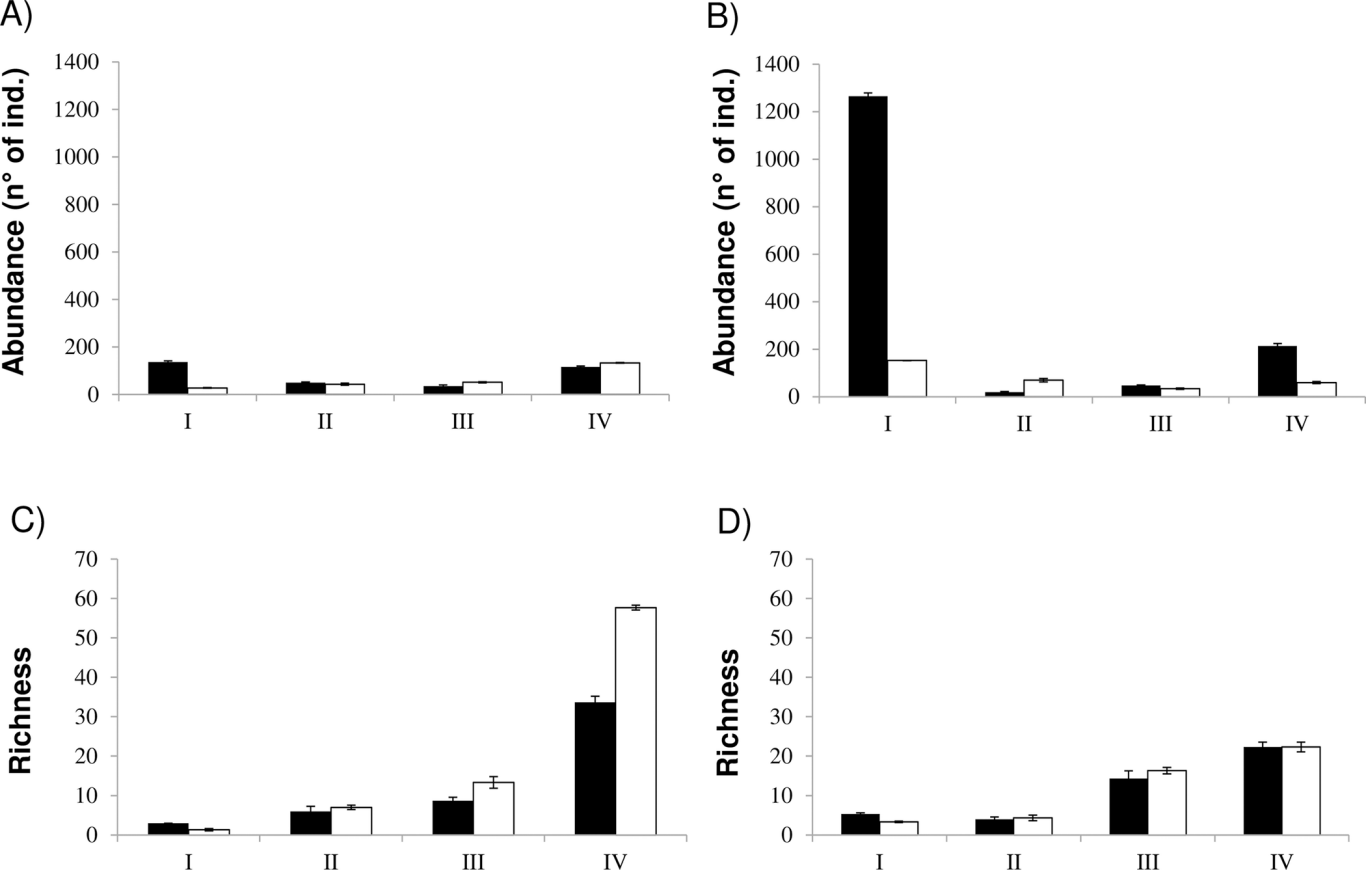
Mean abundance (± standard error) (A; B) and taxon richness (± standard error) (C; D) of benthic macroinvertebrate communities in zones I, II, III and IV of Paraíba do Norte (A; C) and Mamanguape (B; D) estuaries. Black and white bars represent dry wet seasons.

The composition of the benthic macroinvertebrate communities was similar in both estuaries in the dry season (PerMANOVA: F_1,22_ = 1.45; p = 0.14) and wet season (PerMANOVA: F_1,22_ = 1.70; p = 0.09). However, significant differences among zones of the estuarine gradient were found in both periods in the Paraíba do Norte (dry–PerMANOVA: F_3,8_ = 3.92, p = 0.001; wet–PerMANOVA: F_3,8_ = 4.05, p = 0.001) and Mamanguape (dry–PerMANOVA: F_3,8_ = 4.73, p = 0.001; wet–PerMANOVA: F_3, 8_ = 5.21, p = 0.001) estuaries (Fig 3A and 3B).

The richness of the benthic macroinvertebrate taxa in the different zones of the Paraíba do Norte estuary (Fig 3C) was greater during the wet period in all zones (I: 2, II: 15, III: 28 and IV: 80) in comparison to the dry period (I:6; II:13; III:20; IV:52), except zone I. The same situation occurred in the Mamanguape estuary (Fig 3D), which likewise demonstrated greater benthic macroinvertebrate in the wet period (I: 4, II: 10, III: 35 and IV: 45) in comparison to the dry period (I: 10, II: 6, III: 32 and IV: 40).

Taxonomic richness of the benthic communities did not differ significantly between the two seasons (ANOVA: F_1,32_ = 0.02; p > 0.05) or between the two estuaries (ANOVA: F_2,32_ = 0.058; p > 0.05). However, significant differences were found among the salinity gradient zones of the estuaries (ANOVA: F_3,32_ = 45.52; p < 0.05) (Fig 3C and 3D).

### Partitioning of diversity and spatial effects

The greatest dissimilarity of the benthic macroinvertebrate communities occurred in both seasons among estuarine zones (β_3,_ dry = 58.6%; wet = 46.3%) and between estuaries (β_4,_ dry = 17.1%; wet = 27.5%). The β_3_ and β_4_ scales demonstrated higher mean richness values than the values expected solely by chance (Prop_exp>obs_ < 0.001) in both sampling periods (Table 1). On the other hand, observed richness for the lowest hierarchical levels (sampling units, α scale; among sampling units, β_1_ scale; among points, β_2_ scale) was less than that expected just by chance (Prop_exp>obs_ > 0.999). The proportions of variation were respectively 8.4, 6.4 and 9.5% of the total diversity in the dry period and 11.5, 5.9 and 8.7% in the wet period. Gamma diversity was more representative in the wet season (Table 1).

**Table 1:** Observed and expected values generated from additive partitioning in wet and dry seasons in Paraíba do Norte and Mamanguape estuaries, Brazil.

	Dry			Wet		
Scales	Obs	Exp	Prop_exp>obs_	Obs	Exp	Prop_exp>obs_
Diversity of replicates (α)	5.51	37.37	>0.999	11.87	31.09	>0.999
Diversity among replicates (β_1_)	4.18	9.94	>0.999	6.18	18.63	>0.999
Diversity among points (β_2_)	6.28	8.43	>0.999	9.09	18.80	>0.999
Diversity among zones (β_3_)	38.70	6.73	<0.001	48.22	23.32	<0.001
Diversity between estuaries (β_4_)	11.33	3.53	<0.001	28.64	12.16	<0.001
Total diversity (γ)	65			104		

### Turnover and nestedness of benthic macroinvertebrate communities

**Fig 4:**
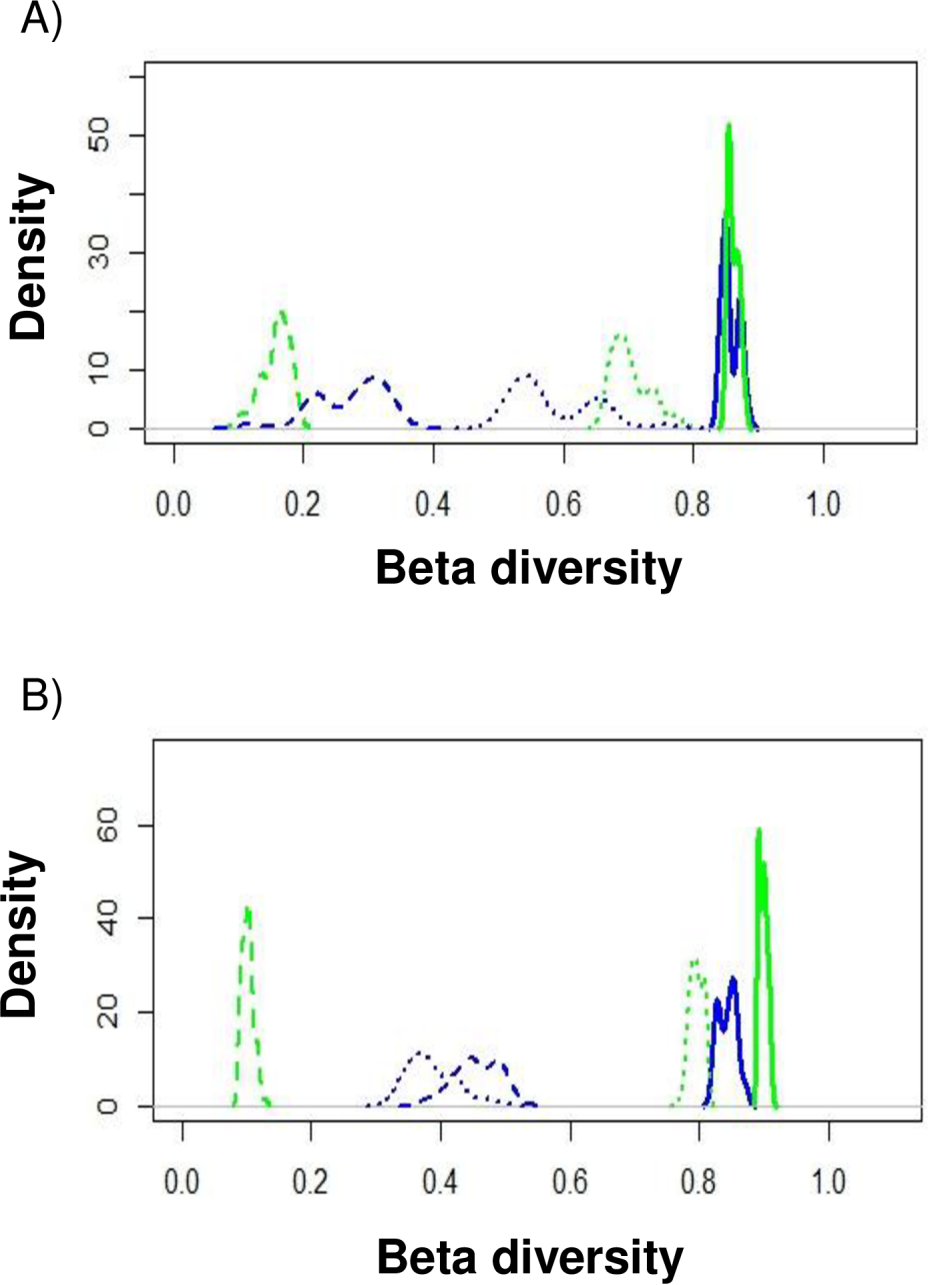
Beta diversity partitioning of turnover and nestedness components in dry (A) and wet (B) seasons. Blue and green shading represent Paraíba do Norte and Mamanguape estuaries, respectively. Continuous lines represent general beta diversity. Dashed and dotted lines represent nestedness of species and turnover.

In the dry season, the Paraíba do Norte and Mamanguape estuaries had similar general beta diversity, which was generated by turnover in both estuaries (Fig 4A). In the wet season, the proportion of general beta diversity was more representative in the Mamanguape estuary and was mainly driven by turnover (Fig 4B), whereas the turnover and nestedness components contributed similarly to general beta diversity in the Paraíba do Norte estuary (Fig 4B).

## Discussion

The estuarine gradient exerted the greatest influence on dissimilarities in the benthic macroinvertebrate communities analyzed (β_3_ scale), with greater proportions of variability on the highest hierarchical levels (scales β_3_ and β_4_) in both seasons, which confirms our first hypothesis. However, our second and third hypotheses were only partially confirmed, as the communities were mainly substituted (turnover) in both seasons in the estuarine system subjected to a lower degree of anthropogenic influence, as expected. In contrast, the benthic macroinvertebrate communities were similarly structured by turnover in the dry season as well as both turnover and nestedness in the wet season in the estuary with the greater degree of anthropogenic influence (Paraíba do Norte).

The salinity gradient acted as an environmental filter for the macroinvertebrate communities in each zone, sustaining only those species with functional or phenotypic traits adequate for establishment and persistence [54, 55]. Thus, the selection of organisms in the various estuarine zones could be explained by their ability to regulate internal ion concentrations. In dispersing to areas with other levels of salinity, the regulation of internal ion concentrations would become exhaustive in terms of energy expenditure and individuals could perish due to a lack of appropriate morphological and/or anatomical adaptations or could be forced to diminish reproductive and developmental rates [56]. Likewise, Bleich *et al*. [57] found that species variation occurred mainly along the salinity gradient in the Baltic Sea and Josefson and Göke [10] also found greater beta diversity among different zones of the salinity gradient in a temperate estuary. The greater dissimilarity on the highest hierarchical levels, as observed among zones and between estuaries in the present study, with observed proportions of variation greater than those expected by chance, suggests that dispersal was limited on these scales in comparison to lower hierarchical scales.

The benthic macroinvertebrate communities in the estuaries analyzed were represented by polychaetes, mollusks and dipterans, which, in response to species selection, exhibited spatial structure reflected on the highest hierarchical levels. Zone I of the estuaries was populated mainly by *Polypedilum* and *Laeonereis*, while the greatest species richness was found in zone IV. The greater richness in zone IV was due to the adaptation of species of the benthic macroinvertebrate community to the environmental conditions of this zone, as higher salinity and a greater variation in sediment particle size allow more species to cohabitate than conditions of low salinity and muddy sediment. Moreover, some larvae and juveniles may also be transported to the estuary through tidal activity [57]. When adults, however, most of these specialist species would be dispersed by tidal mover to areas with low salinity and would not reproduce under such conditions, since they are incapable of generating the high levels of energy necessary for osmoregulation to maintain their vital functions [57]. Consequently, dipterans were only encountered in zones I and II of the estuaries and the taxonomic richness of the communities differed only along the estuarine gradient.

Our results are in agreement with findings described in previous studies that demonstrate a linear relationship between species richness and salinity (upstream to downstream areas) [10, 20, 25, 57, 58]. This pattern of diminishing species richness is explained by the theory proposed by Remane [26], who investigated the distribution of the community along the salinity gradient in the Baltic Sea: the macroinvertebrate community attained greatest richness in the estuarine region with the highest salt concentration, with a reduction in richness accompanying the reduction in salinity along the gradient until the region denominated “*Artenminimum*”, where the salinity levels were from 5 to 8. Richness began to increase again with the greater influence of freshwater input and the appearance of species from freshwater environments.

The variability in community composition between the two estuaries was probably related to differences in the use and occupation of these areas by humans [25], as the Paraíba do Norte estuary is surrounded by urban agglomerations, while the Mamanguape estuary is within an environmental protection area and is surrounded by mangrove forests, which tend to attenuate anthropogenic impacts. Anthropogenic influences have been recognized as some of the main causes of alterations in ecosystem processes and the composition of aquatic estuarine habitats [59, 60]. Although some species were found in both estuaries (e. g., *Laeonereis* and *A*. *brasiliana*), the distribution patterns of species along the estuaries differed in response to differences in the degree of anthropogenic impact, leading to either the substitution of species (turnover) or the formation of nested subsets of species (nestedness). Analyzing the relationship between beta diversity and environmental disturbances, Barros *et al*. [25] proposed a conceptual model demonstrating that ecological conditions in estuaries influence the partitioning of the general beta diversity of benthic macroinvertebrate between turnover and nestedness. In the present investigation, the components of beta diversity in the two tropical estuaries were dependent not only on the intensity of anthropogenic influences, but, as naturally stressed ecosystems, structuring through turnover or nestedness was also influenced by the estuarine gradient and seasonal variations that act as environmental filters. These factors influence passive dispersal, producing variations in the turnover or nestedness components.

The selection of the macroinvertebrate community by the estuarine gradient may have driven the substitution of species in the Mamanguape estuary (with its lower degree of anthropogenic influence) in both seasons as well as in the Paraíba do Norte estuary (with its greater degree of anthropogenic influence) mainly in the dry season, with specific taxa being encountered in the different zones along the salinity gradient [25]. Since the Paraíba do Norte estuary experiences strong anthropogenic impact, the macroinvertebrate community is composed of both specialist and generalist species [25]. Thus, the freshwater input in the wet season favors the drift of generalist species, which are widely dispersed throughout the ecosystem, resulting in a nestedness pattern. Specialist species are selected by the estuarine gradient and are consequently substituted. In this case, the species distribution pattern is the result of both anthropogenic influences and environmental filters.

The temporal scale considered in the present study (dry and wet seasons) was fundamental to the structuring of the benthic macroinvertebrate communities, resulting in significant differences in species abundance. Variations in β and γ diversity were also strongly influenced by season. Thus, the rainfall pattern in tropical estuaries that receive drainage from temporary rivers should also be considered a filter for benthic communities.

General patterns of diversity within and between estuaries have been demonstrated in a robust manner in temperate regions [10, 27, 61]. However, little attention has been given to tropical regions [25]. Thus, a better understanding of the processes by which beta diversity is created and maintained is essential to understanding the structuring mechanisms of these communities and developing conservation strategies that can help to establish priority conservation areas [62].

Our results demonstrate that benthic macroinvertebrate communities in tropical estuaries are mainly structured by species substitution due to the estuarine gradient and the influence of seasonality, which acts as an environmental filter with regard to species selection and drift. The salinity gradient in the two tropical estuaries exerted a strong influence on dissimilarities in the benthic macroinvertebrate communities, with the spatial structure reflected on the highest hierarchical levels and a linear relationship found between species richness and the estuarine gradient from upstream to downstream areas.
